# Multi-scale entropy assessment of magnetoencephalography signals in schizophrenia

**DOI:** 10.1038/s41598-024-64704-2

**Published:** 2024-06-25

**Authors:** E. J. Wolfson, T. Fekete, Y. Loewenstein, O. Shriki

**Affiliations:** 1 Department of Cognitive and Brain Sciences, Ben Gurion University of the Negev, 1 Ben-Gurion Blvd., Beer-Sheva, Israel; 2 The Edmond & Lily Safra Center for Brain Sciences, Department of Cognitive and Brain Sciences,The Alexander Silberman Institute of Life Sciences and The Federmann Center for the Study of Rationality, The Hebrew University, Jerusalem, Israel

**Keywords:** Neuroscience, Schizophrenia

## Abstract

Schizophrenia is a severe disruption in cognition and emotion, affecting fundamental human functions. In this study, we applied Multi-Scale Entropy analysis to resting-state Magnetoencephalography data from 54 schizophrenia patients and 98 healthy controls. This method quantifies the temporal complexity of the signal across different time scales using the concept of sample entropy. Results show significantly higher sample entropy in schizophrenia patients, primarily in central, parietal, and occipital lobes, peaking at time scales equivalent to frequencies between 15 and 24 Hz. To disentangle the contributions of the amplitude and phase components, we applied the same analysis to a phase-shuffled surrogate signal. The analysis revealed that most differences originate from the amplitude component in the δ, α, and β power bands. While the phase component had a smaller magnitude, closer examination reveals clear spatial patterns and significant differences across specific brain regions. We assessed the potential of multi-scale entropy as a schizophrenia biomarker by comparing its classification performance to conventional spectral analysis and a cognitive task (the n-back paradigm). The discriminative power of multi-scale entropy and spectral features was similar, with a slight advantage for multi-scale entropy features. The results of the n-back test were slightly below those obtained from multi-scale entropy and spectral features.

## Introduction

Schizophrenia (SCZ) is a severe disruption of cognition and emotion, affecting the most fundamental human functions: language, thought, perception, affect, and sense of self^1^. Traditionally, its major symptoms have been divided into positive, negative and cognitive symptoms. Positive symptoms are those that appear to reflect an excess or distortion of normal functions such as excitement, hyperactivity, grandiosity etc. Many positive symptoms are related to perceptual distortion and aberrant sensory inputs. In a severe form, these symptoms manifest as illusions, hallucinations, and delusions, however, they may also manifest in various other forms like suspiciousness, and hostility.

Negative symptoms are those that appear to reflect a diminution or loss of normal functions. The most prominent negative symptoms include flattened affect, emotional and social withdrawal, disorganized thinking, poverty of speech (Alogia), difficulties in concentrating, poor rapport and apathy. Cognitive symptoms include somatic concern, anxiety, tension, motor retardation, disorientation, mannerisms and more^2^. Although usually, the positive and negative symptoms are the ones which stand out, almost all SCZ patients exhibit cognitive dysfunction to some degree. This occurs in the domains of attention, executive function, working memory, episodic memory, etc.

During the last 3 decades there have been many attempts to find a biomarker, based on pathologies that characterize brain dynamics that can differentiate SCZ patients from healthy controls. Many of these studies utilized electrophysiological methods such as EEG and MEG, due to their high temporal resolution, which enables capturing to some extent the temporal and spatial complexity of brain activity.

Many electrophysiological studies reported that abnormal neural oscillations play a central part in the complex etiology of SCZ. Most of the studies were focused on the abnormal characteristics extracted from EEG/MEG resting state recordings^3–6^. Furthermore, studies by Linkenkaer-Hansen et al.^7,8^, and Nikulin and Brismar^9,10^, have shown an existence of Long-Range Temporal Correlations (LRTCs) in the amplitude dynamics of α and *β* oscillatory activity across different time windows. Nikulin et al. also reported a significant attenuation in LRTCs related to α and *β* power activity in SCZ patients^11^.

Another natural target for these biomarkers is Complexity estimators extracted from electrophysiological records. Several noteworthy complexity estimators are the Correlation Dimension (D2)^12^, Lyapunov Exponent (L1)^13^, Mutual Information (MI)^14^, Higuchi Fractal Dimension (HFD)^15^, Omega Complexity (CΩ)^16^, and Lempel–Ziv Complexity (LZC)^17,18^. Goshvarpour suggested^19,20^ a combination of 3 nonlinear estimators, complexity (Cx), HFD, and L1, for SCZ diagnosis.

The link between SCZ and complexity is not obvious. Naively, we may relate SCZ with chaos^21^. That is because both positive and negative SCZ symptoms are associated with disorder, unpredictability, and confusion. For instance, disorganization, alogia, concentration difficulties etc. might be associated with noisy systems, while hallucinations and delusions seem to be related to chaos or unpredictability. However, the term complexity itself is not well defined, and the scientific notion of it often depends on the field in which it is applied. One common definition for complexity, introduced by Neil Johnson, is *“the study of the phenomena which emerge from a collection of interacting objects”*. Therefore, the association between SCZ and disorder is mostly based on behavioral observations rather than mechanistic modeling and reflects many social and cultural aspects. Furthermore, previous studies employing different complexity estimators often yielded ambiguous findings as to whether SCZ patients have increased or decreased complexity in comparison to healthy controls. For instance, several studies utilizing the D2 estimator derived from EEG records, reported increased complexity in SCZ patients^16,22,23^, while, other studies reported reduced complexity in SCZ patients based on a similar methodology^24,25^. Another study using the D2 estimator for sleep EEG monitoring^26^, reported both increased and reduced complexity in SCZ patients depending on sleep stage. Alberto Fernández in a review paper from 2013^21^ summarized all the complexity studies in SCZ patients and argued that a substantial part of this ambiguity might be attributed to factors like medication effects, symptomatology, age, and even social background. However, the vague notion of what complexity is also contributes to the variable and contradictory findings.

Entropy-based estimators have been proven as efficient and robust for evaluating EEG/MEG regularity or predictability. In *The Theory of Communication*, Claude Shannon borrowed the term *entropy* to quantify the rate of information carried over a communication channel^27^. However, conventional methods to assess entropy of a time series are not well suited to noisy and relatively short data, as frequently encountered in biological studies. In an attempt to utilize information entropy for biological systems, Steven Pincus introduced Approximate entropy (ApEn)^28–30^—a complexity measure closely related to entropy, that can be easily applied to biological and clinical data. Later, it was elaborated upon in Sample Entropy (SampEn)^31^, which according to the authors, proved to be a more consistent method in terms of results, whilst keeping the general essence of the original information entropy as defined by Shannon. Finally, Costa, Goldberger, and Peng^32–35^ expanded the SampEn into MSE (MultiScale Entropy), by adding the element of computing the SampEn across different scales. In this study, we utilized the MSE approach to assess the complexity of the information carried by the reported LRTCs.

The potential of developing a biomarker from SampEn and MSE is related to the fact that entropy-based methods are sensitive to both the phase and the amplitude dynamics of neural oscillations, unlike conventional Power Spectral Density (PSD) analysis which reveals only amplitude dynamics. Miskovic et al.^36^ used phase-shuffled surrogates to demonstrate that changes in EEG complexity across the sleep cycle cannot be solely explained by linear dependencies (i.e., PSD) but also involve phase dynamics. Several studies have supported the potential of entropy-based biomarkers. For instance, multiple MSE studies in Alzheimer's^37–39^ identified significant differences between patients and control subjects over large time scales, showcasing classification capabilities with sensitivity and specificity around 80%, and an area under the ROC curve of 0.83. Bosl et al.^40^ suggested that MSE extracted from resting-state EEG data could be a useful early detection biomarker for ASD (autism spectrum disorder) risk and for identifying cognitive development abnormalities in infants. Additionally, a study by Catarino et al.^41^ found a significant difference in SampEn, related to task-specific information processing, between ASD patients and neurotypical controls.

### Previous studies in SCZ

Previous studies on schizophrenia have applied the MSE analysis to evaluate patients. Takahashi et al.^42^ conducted one of the most notable studies, recording resting-state EEG from 22 SCZ patients and 24 healthy controls. Their study showed that SCZ patients generally exhibited a significantly higher Sample Entropy at higher time scales than controls. Similarly, Sabeti et al.^43^ and Brookes et al.^44^ found significantly increased task-induced SampEn in SCZ patients.

However, the robustness of these results is questionable, due to the relatively small number of participants in both studies (*n* = 22 & 20 for Takahashi and Sabeti studies, respectively), and the variable nature of complexity estimators. Takahashi addressed the medication factor in his study by testing the effects of neuroleptic medication on MSE values (one of very few studies to do so). All the 22 SCZ patients were recorded during a pre-treatment onset phase. Fifteen of the patients were re-evaluated 2–8 weeks after the initiation of the antipsychotic drugs treatment. The pre-treatment MSE values were substantially higher compared to normal controls. Post-treatment MSE values decreased yet remained significantly higher than control values. However, focusing on drug naïve patients naturally narrows the age range of the patients, and indeed the mean patients’ age in Takahashi’s study was quite young (25 ± 4.8). In Sabeti’s study all the patients were already treated with neuroleptic medication, so the age range was wider (33 ± 10) and more representative.

Other studies have explored closely related methods. Xiang et al.^45^ applied Fuzzy entropy (FuzzyEn) to EEG signals recorded during auditory tasks. Bai et al.^46^ studied Multiscale Weighted Premutation Entropy analysis (MSWPE) in MEG signal acquired in resting state from SCZ patients and controls. Both studies identified significant abnormalities in the signals acquired from SCZ patients compared to healthy controls.

Takahashi and Sabeti's studies suggest that MSE analysis might provide more neurophysiological information than traditional power spectrum analysis, offering greater discriminative power. However, both studies primarily relied on comparison of experimental results. From a computational standpoint, it remains unclear whether MSE analysis captures additional information—stemming from the ordering of long-range temporal correlations (LRTCs)—beyond that manifested in the power spectrum. This additional information might be hidden in the phase component of the time series^36^. In this study, we addressed this question explicitly, by applying a phase shuffling algorithm to the original time series, in order to disentangle the contribution of the linear order (amplitude component) from the contribution of the long-range temporal correlations (phase component) to the MSE values. We also examined the nature of the phase component to determine if the information it contains offers discriminative and thus classificatory potential.

## Methods

### Participants

This study included 49 patients and 98 healthy controls. Patients included 33 males and 16 females with a mean age of 33.5 ± 1.8 years, out of which 3 were left-handed. The control group included 62 healthy females and 36 males with a mean age of 32.4 ± 1.1 years, out of which 7 were left-handed. All the patients met DSM-IV criteria for schizophrenia or schizotypal disorder and were treated with antipsychotic medications. Controls did not have any neurological or psychiatric illnesses or any history of head trauma. All the controls underwent a Structured Clinical Interview and were reported normal. All experiments were carried out in accordance with NIH guidelines for human subjects and were approved by the Institutional Review Board of the National Institute of Mental Health (NIMH, Bethesda, MD, USA). Informed consent was obtained from all subjects and/or their legal guardian(s).

### MEG recording

Data were collected at the MEG core facility of the NIMH. The MEG system (manufactured by CTF Systems, Inc.), comprised 275 axial SQUID gradiometers uniformly distributed over the inner surface of a whole-head helmet. Records of 4 min with sampling frequency (*f*_s_) of 600Hz (144,000 samples in total) in resting-state conditions were obtained from each participant. Two gradiometers were unstable during some of the records and therefore excluded from the analysis. The raw signal was band-pass filtered from 1-80Hz. Direct current offset and power line noise were removed using a minimal high pass filter (0.61 Hz) and a notch filter for power line (60 Hz and harmonics), respectively. Head position was determined, real-time, inside the magnetometer by recording the position of reference coils attached to the nasion and bilateral preauricular points of each participant. All the participants were instructed to minimize eye-movement. Next, an Independent Component Analysis (ICA) decomposition was applied to each dataset to reject artifacts such as heartbeats (ECG) and noise from skeletal muscles (EMG).

### Multi scale entropy (MSE) analysis

The MSE method^32,33^ quantifies the degree of regularity within a time series by evaluating repetitive sequences of data points across different timescales. For a given time scale, data points are considered to be indistinguishable if the absolute difference between them is smaller than the tolerance value r (r typically varies between 0.1 and 0.25 times the standard deviation of the time series’). Sequences, in turn, are considered to match each other if every point in the first sequence is indistinguishable from the corresponding point in the matched sequence. For a given time series with *N* data points, the Sample Entropy (SampEn) is defined as:

1 $$SampEn\left( {m,r} \right) = \mathop {\lim }\limits_{N \to \infty } - {\text{ln}}\frac{{C^{m + 1} \left( r \right)}}{{C^{m} \left( r \right)}}$$

where ${C}^{m}$ and ${C}^{m+1}$ are the total number of sequences matches with *m* and *m* + 1 points correspondingly, excluding self-matches. For a finite *N*, it can be estimated as:

2 $$SampEn\left( {m,r,N} \right) = - {\text{ln}}\frac{{C^{m + 1} \left( {r,N} \right)}}{{C^{m} \left( {r,N} \right)}} = \ln \frac{{\mathop \sum \nolimits_{i = 1}^{N - m} C_{i}^{m} }}{{\mathop \sum \nolimits_{i = 1}^{N - m} C_{i}^{m + 1} }}$$

In qualitative terms, the SampEn for each time scale is equal to the negative natural logarithm of the conditional probability that sequences matching each other for *m* consecutive data points, will remain matched at the next point (*m* + 1). (For further details, please refer to Costa et al.)^32,33^

The different time scales are obtained by coarse graining the original time series into non-overlapping time windows and then averaging the data points inside each time window as follows:

3 $$y_{j}^{\left( \tau \right)} = \frac{1}{\tau }\mathop \sum \limits_{{i = \left( {j - 1} \right)\tau + 1}}^{j\tau } x_{i} , 1 \le j \le \frac{N}{\tau }$$

The number of the original samples in each window is called the Scale Factor (SF) *τ*. This process of coarse graining is a form of down-sampling; therefore, each scale can be translated into the Equivalent Sampling Rate (ESR) by:

4 $${\text{ESR}}\left( {f_{s} } \right) = \frac{1}{{f_{s} }}$$

Considering *f*_s_ = 600 HZ, scale factors of 4 and 10 will be equivalent to sampling at 150 Hz and 60 Hz, respectively. Furthermore, according to the Nyquist theorem, the maximum sample frequency is half of the sampling rate, therefore the SF = 4 & 10 will sample frequencies from 0 to 75 & 0 to 30 Hz correspondingly. In our case, since the data went through a high pass filter, the lowest frequency is 0.6 Hz.

To optimize both accuracy and time efficiency, we calculated 38 scale-factors from 4 to 120 (1200 samples for SF = 120). While there are no definitive rules for determining the r and m parameter; several theoretical and clinical studies^32,34,47,48^ have demonstrated that m = 1 or 2, and r = 0.1–0.25, provides good statistical validity. During our preliminary analysis with a subset of the data, we examined tolerance values r from 0.07 to 0.23. The most consistent and smooth results were obtained for *r* = 0.135–0.2 in agreement with the literature^35^. For the final analysis, we used m = 2, r = 0.2 to keep consistency with previous studies^39,42,43^.

To compare the SampEn of two groups, we introduce the ΔSampEn which is simply the difference between two SampEn values normalized by their average:

5 $${\Delta }SampEn = 2 \frac{{SampEn_{{{\text{SCZ}}}} - SampEn_{{{\text{NC}}}} }}{{SampEn_{{{\text{SCZ}}}} + SampEn_{{{\text{NC}}}} }}$$

To further quantify the SampEn with respect to a scale region, we introduced the *κ* parameter which is simply the mean of the SampEn function in each region of scales:

6 $$\kappa = \frac{1}{{x_{2} - x_{1} }}\int\limits_{{x_{1} = SF_{1} }}^{{x_{2} = SF_{2} }} {SampEn\left( x \right)dx}$$

**Figure 1:**
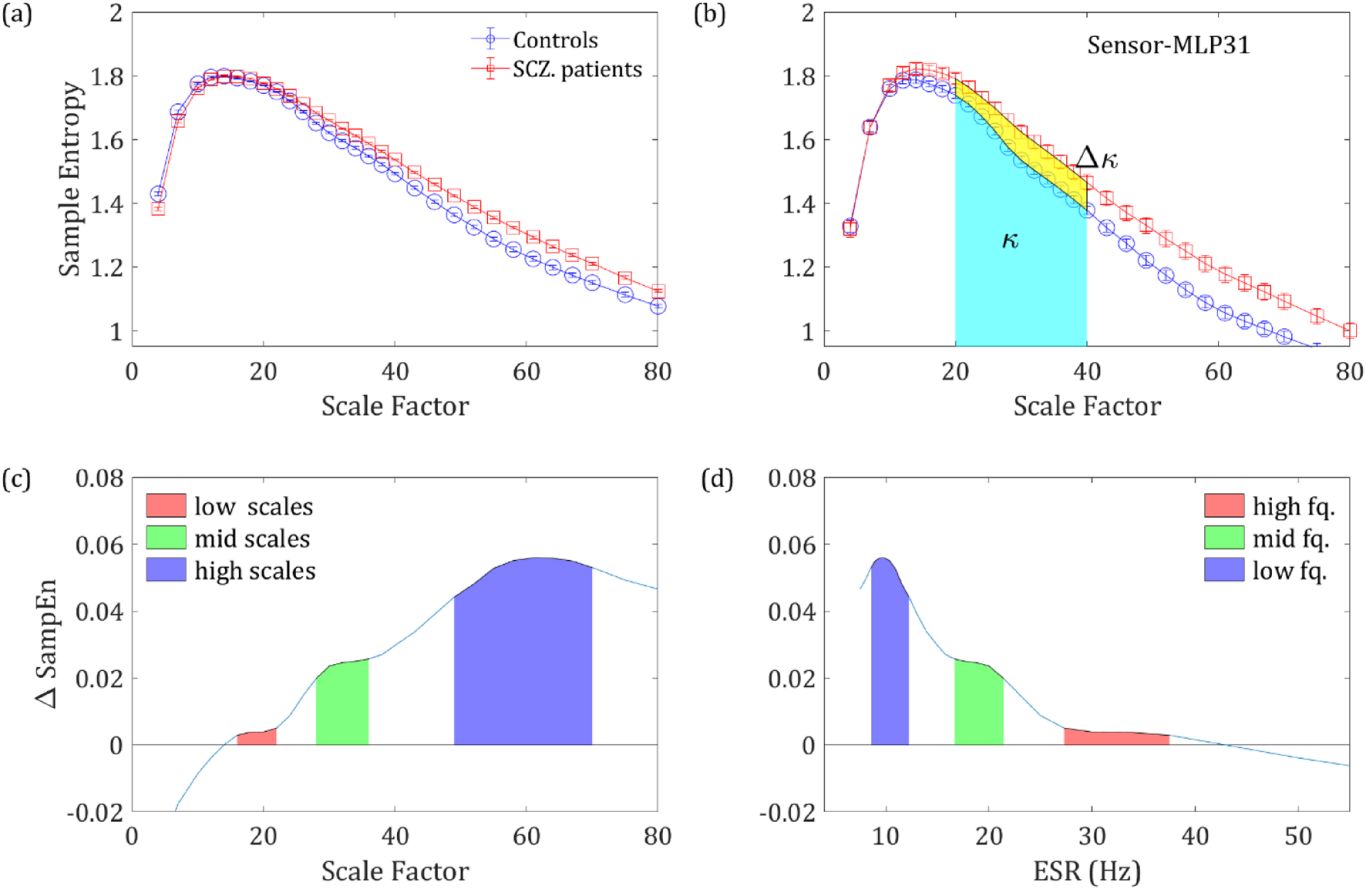
Differences in SampEn between the two groups. (**a)** SampEn vs. scale factor, grand average of the controls (in blue) and SCZ (red) across all sensors. **(b)** The grand average of a single representative sensor (belonging to the 90 percentile of t values obtained via univariate *t*-tests). The measure *κ* is defined as the mean SampEn in a given scale region (area below the curve, divide by the length of the region) (20–40 in this example; cyan shading), whereas Δκ is the difference between the mean values of the two groups (yellow shading). **(c)** The difference between controls and SCZ vs. scale factor. The colored areas represent 3 regions in which the difference peaked, red–low scales, green–mid scales and blue–high scales. **(d)** The same difference plotted against the frequency corresponding to the scale factor (ESR), the colored areas correspond to the regions in plot c.

Similarly, Δ*κ* is defined as the difference between the *κ* values of the two groups in the same region normalized by the average: (see Fig. 1b).

7 $${\Delta }\kappa = 2\frac{{\kappa_{{{\text{SCZ}}}} - \kappa_{{{\text{NC}}}} }}{{\kappa_{{{\text{SCZ}}}} + \kappa_{{{\text{NC}}}} }}$$

### Phase shuffling analysis

To disentangle the contribution of the amplitude component to the entropy (i.e., the information manifested in the power spectrum) from the contribution of the phase component (i.e., information related to LRTCs), we generated surrogate data from the original time series^49^. This was obtained by Fourier transforming the data, randomly shuffling the phases, and then applying the inverse transform. Subsequently, we applied the MSE analysis to the phase-shuffled data from each participant. To quantify the contribution of the phase component, hence the Non-Linear Component (NLC), we defined *κ*_*nlc*_ for each participant as the difference between the *κ* values of the PSF and the original time series:

8 $$\kappa_{{{\text{nlc}}}} = \kappa_{{{\text{psf}}}} - \kappa_{{{\text{org}}}}$$

The Δ*κ*_nlc_ in turn is defined as the difference in the residue between SCZ patients and controls groups:

9 $${\Delta }\kappa_{{{\text{nlc}}}} = \left( {\kappa_{{{\text{psf}}_{{{\text{SCZ}}}} }} - \kappa_{{{\text{org}}_{{{\text{SCZ}}}} }} } \right) - \left( {\kappa_{{{\text{psf}}_{{{\text{NC}}}} }} - \kappa_{{{\text{org}}_{{{\text{NC}}}} }} } \right)$$

While Δ*κ’*_nlc_ is defined as the normalized difference.

10 $${\Delta }\kappa ^{\prime}_{{{\text{nlc}}}} = 2\frac{{\kappa_{{{\text{nlc}}_{{{\text{SCZ}}}} }} - \kappa_{{{\text{nlc}}_{{{\text{NC}}}} }} }}{{\kappa_{{{\text{nlc}}_{{{\text{SCZ}}}} }} + \kappa_{{{\text{nlc}}_{{{\text{NC}}}} }} }} = 2\frac{{\left( {\kappa_{{{\text{psf}}_{{{\text{SCZ}}}} }} - \kappa_{{{\text{org}}_{{{\text{SCZ}}}} }} } \right) - \left( {\kappa_{{{\text{psf}}_{{{\text{NC}}}} }} - \kappa_{{{\text{org}}_{{{\text{NC}}}} }} } \right)}}{{\left( {\kappa_{{{\text{psf}}_{{{\text{SCZ}}}} }} - \kappa_{{{\text{org}}_{{{\text{SCZ}}}} }} } \right) + \left( {\kappa_{{{\text{psf}}_{{{\text{NC}}}} }} - \kappa_{{{\text{org}}_{{{\text{NC}}}} }} } \right)}}$$

To test whether the difference in Δκ_nlc_ between two anatomical areas is significant, we interduced the following ratio, *R* for each participant and each tested anatomical area:

11 $$R = \frac{{{\rm K}_{{{\text{psf}}}} }}{{{\rm K}_{{{\text{psf}}}} + {\rm K}_{{{\text{nlc}}}} }}$$

where *Κ* is the averaged Δ*κ* across all sensors within the given area. We futher interduced *ee’* and ΔR as the ratio and the difference between *R* of two anatomical areas *R*_1_ and *R*_2_.

12 $$R^{\prime} = \frac{{R_{1} }}{{R_{2} }} \,and\, {\Delta }R = R_{1} - R_{2}$$

The *t*-test were performed between the values of *R* ‘and Δ*R* of SCZ patients and controls.

### Classification

We used two different classification methods: Linear Discriminant Analysis (LDA) with lasso (Least Absolute Shrinkage and Selection Operator) regularization for shrinkage^50,51^ and Gradient Boosting. Gradient Boosting is a classification tree which utilizes several weaker classifiers in successive stages to reach the final decision^50^.

### Power spectrum analysis

The Power Spectrum Density (PSD) was computed for each participant and each sensor then averaged across participants and across the relevant scalp areas. The following power bands were extracted: *δ* (0.5-4 Hz), *θ* (4–7.5 Hz), *α* (7.5–12.5 Hz), *β* (15-30 Hz), and *γ* (30-40 Hz). Normalized PSD was used to reduce the impact of individual differences in absolute power.

### n-back task

The n-back task, originally introduced by Kirchner (1958)^52^, is a continuous-recognition measure, in which participants are presented with a stimulus sequence, such as letters or numbers. For each item in the sequence, the participant should decide whether it matches the item presented *n* items back. For instance, in an auditory task, the following sequence of numbers might be played to the test subject:

$$1\;3\;2\;{\mathbf{5}}\;{\mathbf{2}}\;{\mathbf{5}}\;\underline{5} \;3\;6\;4\;3\;{\mathbf{4}}\;\underline{4} \;1\;\underline{{\mathbf{4}}} \ldots$$

In 2-back task the subject should indicate when the number marked in bold is played, because those correspond to the number that were played 2 steps earlier. In the 3-back task the subject should indicate the underlined number. The n-back is considered to be a highly challenging task, and a standard “executive” Working Memory (WM) measure Previous studies using n-back tasks, have indicated that SCZ patients preform significantly more poor relative to healthy controls^53–57^. For further reading see Coulacoglou & Saklofske^58^.

The study included blocks of *n* = 0, 1 and 2 steps back tasks. The data included the score and reaction time form 97 controls and 49 of the patients. We correlated the n-back results with our findings to get an idea of the relationship between computational biomarkers and cognitive impairment.

### Software

All the pre-processing was carried out in a MATLAB environment using the EEGLAB toolbox^59^. The MSE analysis was implemented in MATLAB and is available on GitHub. The LDA classifier and the analysis of the n-back task were also implemented in MATLAB. The gradient boosting was implemented in python using XGBoost open-source library.

## Results

Figure 1a depicts the grand average SampEn across subjects and sensors as a function of the scale factor. Consistent with previous studies, the SampEn was significantly higher in SCZ patients compared to controls^16,42,43^. A higher entropy was observed in 216 of 273 channels. The grand average of a single representative sensor is depicted in Fig. 1b, which also illustrates the definition of the measure κ as the mean SampEn in a given scale region (see Methods). To test the significance of the effect, we conducted a *t*-test of the *κ* values extracted from the SF ranging from 4 to 80, ‘full range’ in Table 1. The effect reached statistical significance (p < 0.05) in 124 channels and 96 channels remained significant after False Discovery Rate (FDR) adjustment (*q*-value < 0.05). Notably, the difference emerged around the scale factor 10 (corresponding to a frequency of 60 Hz; See Materials and Methods) and persisted throughout the range up until the highest calculated scale factor 80 (*f* = 7.5 Hz). The difference peaked around scale factor 60 (*f* = 10 Hz), with two other local maxima at around scale factors 34 and 22 (Fig. 1c, d). For subsequent analyses, in addition to the ‘full range’ we define 4 more SF Regions Of Interest (ROI): high scales, middle scales, low scales, and very low scales (VLS), with their definitions and results summarized in Table 1. These scales are equivalent to sampling rates of 150–7.5 Hz which enable detection of signal frequencies of 75–3.75 Hz (due to the Nyquist criterion).

**Table 1:** Scale factors for range analysis.

Range	Scale factors	ESR (Hz)	Sampled freq. (Hz)	*p*-value	*q*-value
Full range	4–80	150–7.5	0.6–75	124	96
High scales peak	48–70	12.5–8.5	0.6–6.25	67	36
Middle scales peak	28–36	21.4–16.7	0.6–10.7	140	111
Low scales peak	16–22	37.5–27.5	0.6–33.75	165	138
Very Low Scales (VLS)	4–12	150–50	0.6–75	14	0

**Figure 2:**
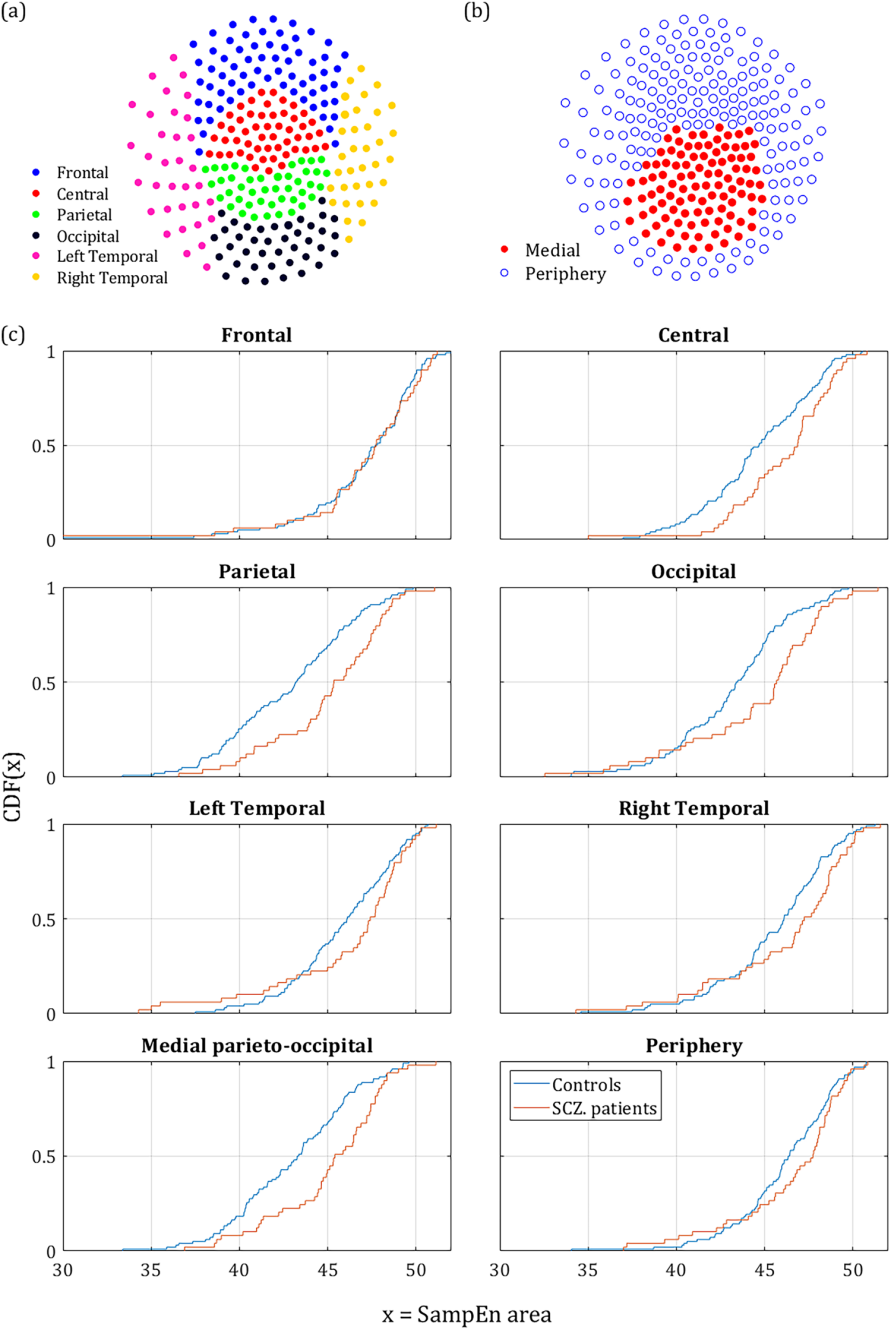
Differences in MSE across different anatomical areas. (**a)** anatomical areas according to conventional EEG/MEG mapping^60^. **(b)** suggested mapping according to our results, dividing the scalp into medial parieto-occipital region and periphery. **(c)** The CDF of κ averaged across different anatomical locations along the scalp. It is noticeable that Parietal Occipital and Central lobes exhibit a more pronounced difference than the frontal and temporal sensors. However, the largest difference was found in the middle of the scalp.

**Figure 3:**
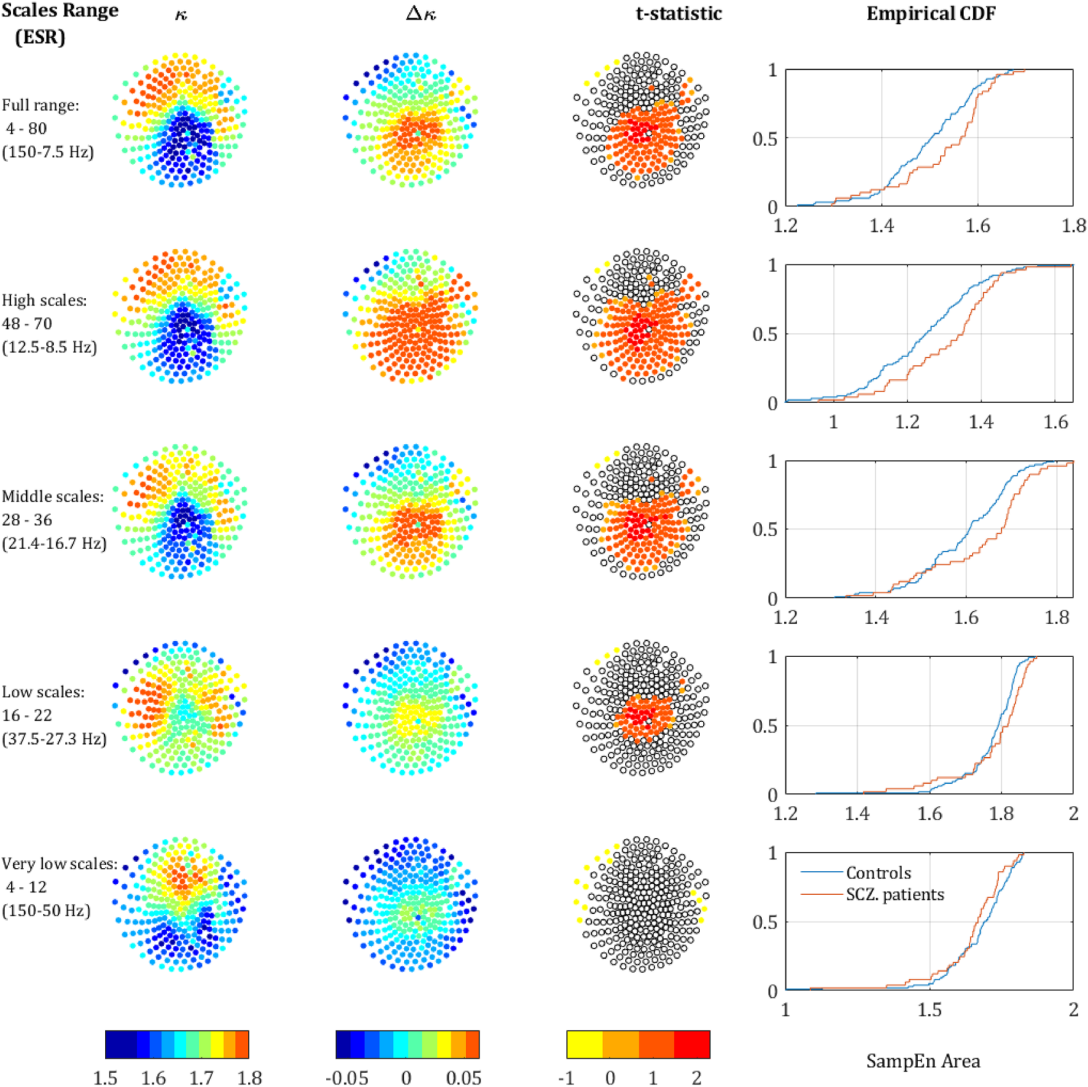
SampEn strength for different scale ranges. The left column depicts the sensors mapping of the *κ* value averaged across both populations (each dot represents a sensor). The second column depicts Δ*κ*, the difference between SCZ and controls. The third column presents the t-statistic value of sensors which reached significance of *p*-value below 0.05 (*t*-test, uncorrected). The rightmost column depicts the CDF curves of the *κ* values in SCZ and controls. Each row represents a different range of scales. Full range (Broadband), high, intermediate, low, and very-low. In brackets is the equivalent sampling rate (ESR).

ESR is the equivalent sampling rate for each analyzed range while the sampled frequency represents the range of recoverable frequencies according to Nyquist theorem of each range (see introduction to this chapter). The *p*-value presents the result of a *t*-test comparing κ values between the two groups extracted from the respective range. The *q*-value column displays the False Discovery Rate (FDR) adjustment applied to the *p*-values,

To further study the anatomical characteristics of the MSE values, we calculated the difference in *κ* between controls and SCZ patients for 6 different scalp areas: Frontal, Central, Parietal, Occipital and Left & Right temporal lobes (Fig. 2a). The values of *κ* were calculated across the full range (4–80 SF). The largest difference was found in the parietal and occipital areas (Fig. 2c). The central area also showed a substantial difference between the groups. The temporal lobes showed a moderate effect and there was practically no effect in the frontal lobe. The results of all SF ROIs and scalp areas are summarized in Table 1 and Fig. 3. To test the robustness of the effect to differences between SCZ and Controls, and to exclude possible biases, we tested the same effect across sex and handedness. The test across sex reached significance in 46 out 273 channels. This result could be expected, since the groups were unbalanced in that respect (67% and 37% males in the SCZ and control groups, respectively). The test for handedness yielded no significant channels at all (See supplementary Information).

Out of the sensors that reached significance, in 120 the *κ* value was higher for the SCZ patient and only 4 sensors showed a higher *κ* for controls. All 4 were located next to each other at the left edge of the frontal cortex between the frontal and left temporal areas (yellow dots in the t-statistic column of the upper row in Fig. 3). The rest of the significant sensors were located mostly in the occipital-parietal cortex with few sensors at the edges of the right and left temporal areas. More careful examination reveals that out of 124 sensors with significant difference in Δ*κ*, 113 sensors were located around the posterior frontal, parietal, and the anterior occipital lobes. Therefore, we suggest dividing the scalp into two regions (Fig. 2b): the medial parieto-occipital region, which shows low SampEn *κ* (high complexity) and high Δ*κ,* and the periphery, in which the SampEn is higher and the difference between groups, Δ*κ,* is much lower.

The upper row in Fig. 3 depicts the *κ* and Δ*κ* across all scales and the corresponding.

t-statistic for each sensor. It is quite noticeable, that the low *κ* values (hence low entropy or high temporal correlations) are concentrated at the back-middle of the scalp (mostly central parietal and occipital). Moreover, the difference between the SCZ patients and controls Δ*κ* also peaks at the center of the scalp and correlates strongly to low *κ.* This can be expected because the areas exhibiting lower entropy (or high temporal correlations) are the areas where one would expect to find a higher difference. The Cumulative Distribution Function (CDF) curves of the *κ* values for controls and SCZ patients is shown in the rightmost column of Fig. 3.

### Scale range analysis

We further analyzed the behavior of the SampEn vs. range of SF. We extracted *κ* for the regions around the 3 peaks that were observed along the difference (ΔSampEn) curve (Fig. 1c,d) and added to it another range of Very Low Scales (VLS), hence high frequencies. The ranges are summarized in Table 1 and the results of the analysis after breaking down to different ranges are depicted in Fig. 3. The analysis shows a clear trend, according to which not only the difference Δ*κ* between SCZ and controls increases with scale, but also the significance as expressed by t-statistics (middle column) and the difference between the Cumulative Distribution Function (CDF) curves of the *κ* values (left column). These results are in good agreement with recent work, which stresses the abnormality found in the *α* and *δ* power bands in SCZ patients^3^.

### Phase-shuffling analysis

To tease apart the contribution of the amplitude component (or the linear component) to the entropy from the contribution of the phase component (the nonlinear component), we generated a phase-shuffled signal from the original time series for each participant and applied the same MSE analysis to it (see methods).

**Figure 4:**
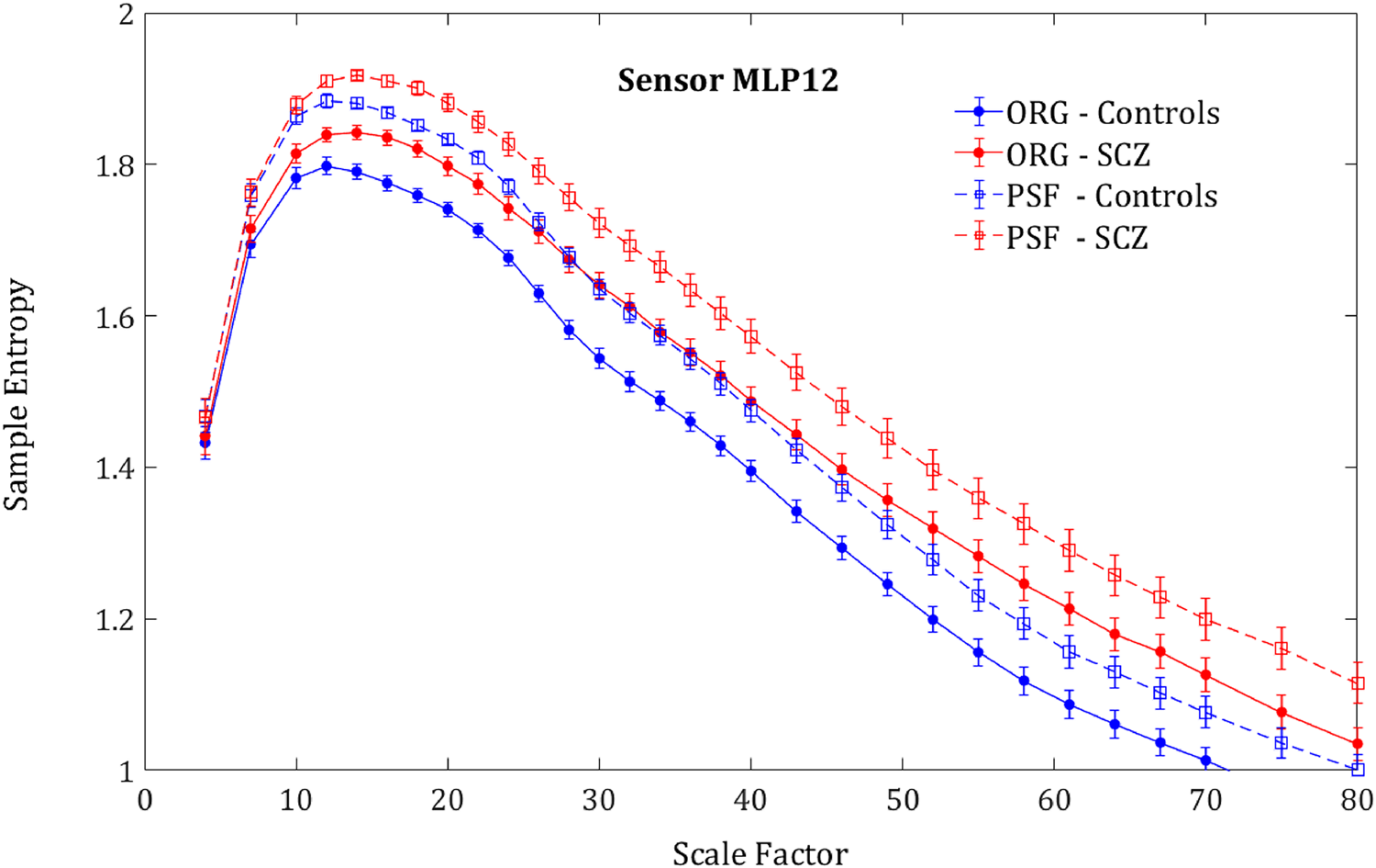
Phase shuffled MSE. SampEn grand averages across groups of MLP12 sensor. Solid lines represent the SampEn of the original signal and the dashed lines the SampEn of the phase shuffled signal. For both groups, the shuffled signal produced noticeably higher SampEn values due to loss of order originating from phase correlations.

**Figure 5:**
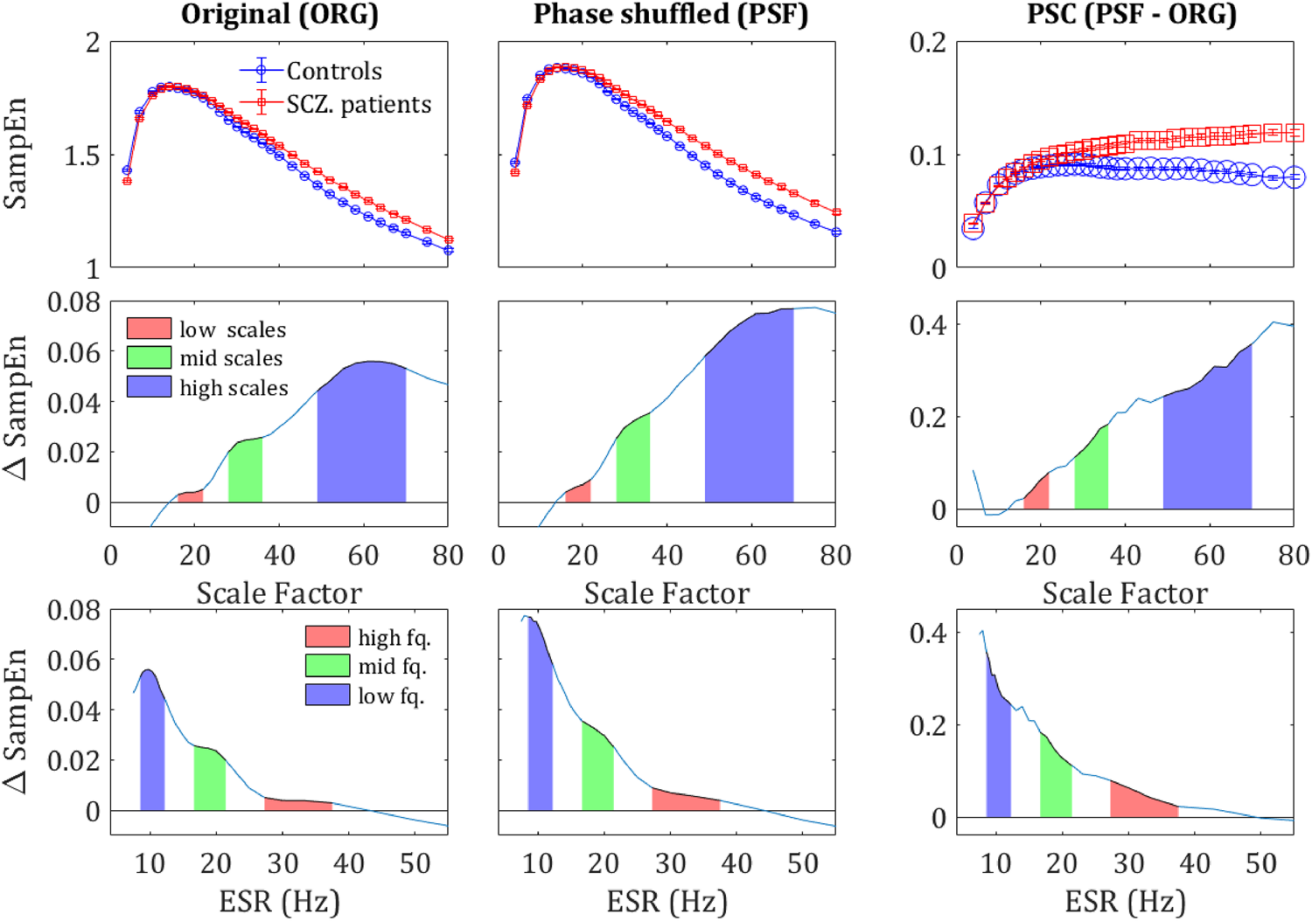
MSE differences by component. The upper row shows the SampEn grand average across all sensors and subjects, for the original signal (left column), Phase shuffled signal (middle column) and the difference between the two (right column) i.e., the phase component. The middle row shows the difference in SampEn between the two curves of the upper plot correspondingly. The lower row shows the same difference plotted against the corresponding frequency (ESR). The colored areas correspond to the regions in Fig. 1 and the values are specified in Table 1.

**Figure 6:**
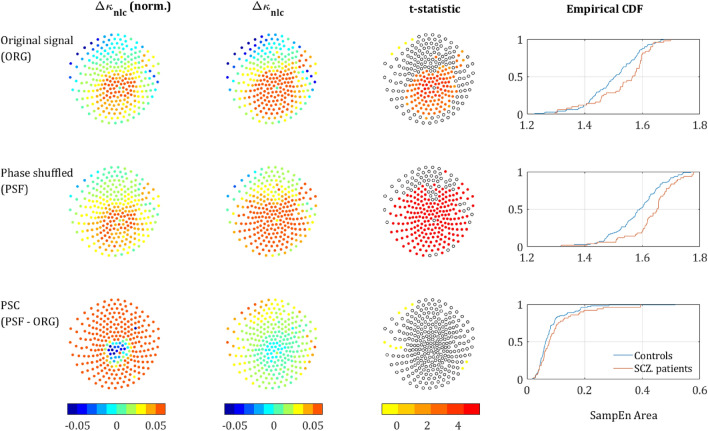
Phase-shuffled MSE across channels. The left column depicts the normalized Δ*κ*_nlc_ between SCZ and controls, each dot represents a sensor. The second column presents the absolute Δ*κ*_nlc_*,* and the third column presents the t-statisic value for sensors that reached significance (p < 0.05, uncorrected). The rightmost column depicts the cumulative distribution function (CDF) of *κ* values averaged in SCZ and controls. The upper row represents the results derived from the analysis of the original signal, the middle row represents the analysis of the phase shuffled signal, and the lower row represents the NLC. The color scale is equal for all 3 Δ*κ* (Original, PSF and NLC) in both the first and second columns.

As expected, the phase-shuffled signal (PSF) yielded a significantly higher SampEn in all.

participants in comparison to the original signal (ORG), indicating a decrease of the order due to loss of long-range temporal correlations (Fig. 4). One could expect that the entropy difference between the groups would diminish due to more substantial order decrease in controls, who had more order in the original signal. However, rather surprisingly, the entropy increment obtained by phase-shuffling was slightly larger for SCZ patients (Figs. 5 and 6) and actually increased the difference between the SCZ patients and controls in contrast with the naïve expectation. This outcome was consistent across all channels and scales.

SampEn extracted from the PSF time series yielded more substantial difference in term of *κ* values than the SampEn extracted from the ORG time series, as can be seen in the CDF curves in the right column in Fig. 6 (note the area enclosed by the two curves). Substantial differences were also found in terms of Δ*κ* as shown in the left column of Fig. 6 and the 2nd row in Fig. 5, and t-statistic as demonstrated by in the 3rd column of Fig. 6.

The *κ* value of the Phase Component (PSC) is defined as the difference between the *κ* values of the PSF time series and the original time series, ${\kappa }_{\text{nlc}}= {\kappa }_{\text{psf}}-{\kappa }_{\text{org}}$_._ For most of the sensors, the difference in *κ*_nlc_ between the groups was not significant (lowest row in Fig. 6). However, when averaging across all sensors and all subjects, this difference was significant. Moreover, it did show a clear trend both across scales and sensors, as can be seen in the right column of Fig. 5 and the low row in Fig. 6. These results indicate that the difference between SCZ patients and controls associated with the order generated by LRTCs might be more substantial than a naïve analysis would predict.

### Analysis of the phase component

It is noticeable that mean Δ*κ*_nlc_ between the groups is much larger than Δ*κ*_psf_ and Δ*κ*_org_ (second and third rows in Fig. 5; note the difference in the scales of the y-axes). This phenomenon is also demonstrated by the sensor mapping (Fig. 6). The sensor mapping also reveals another unpredicted phenomenon, namely the clear spatial trend of the NLC. While the Δ*κ*_nlc_ values in the central ROI are fluctuating around zero, in the periphery Δ*κ*_nlc_ is clearly positive. Yet, Δ*κ*_nlc_ has reached significance only on 10 sensors in the peripheral ROI, due to the small amplitude of the *κ*_nlc_ (rightmost column in Fig. 6) relative to ${\kappa }_{\text{psf}} \text{and} {\kappa }_{\text{org}}$ and fluctuations in their values. To see whether this phenomenon is substantial, we depicted (2nd column in Fig. 6) the mapping of Δ*κ*_nlc_ and Δ*κ’*_nlc_. The mapping of Δ*κ*_nlc_ reveals that the magnitudes of Δ*κ*_psf_ and Δ*κ*_nlc_ have contradicting spatial trends and, actually, look complementary to each other. To test whether this phenomenon is significant, a *t*-test was performed between the values of *R* ‘and Δ*R* of SCZ patients and controls. The difference in both values was found to be significant with p < 0.05 for *R*’ and p < 0.013 for Δ*R.*

### Classification using MSE features

To test the potential of MSE as a biomarker, we designed a classification test to compare the classification potential of MSE features versus conventional features extracted from the Power Spectral Density (PSD). We also compared it to LDA classification performed with 6 features extracted from performance in the n-back paradigm.

**Figure 7:**
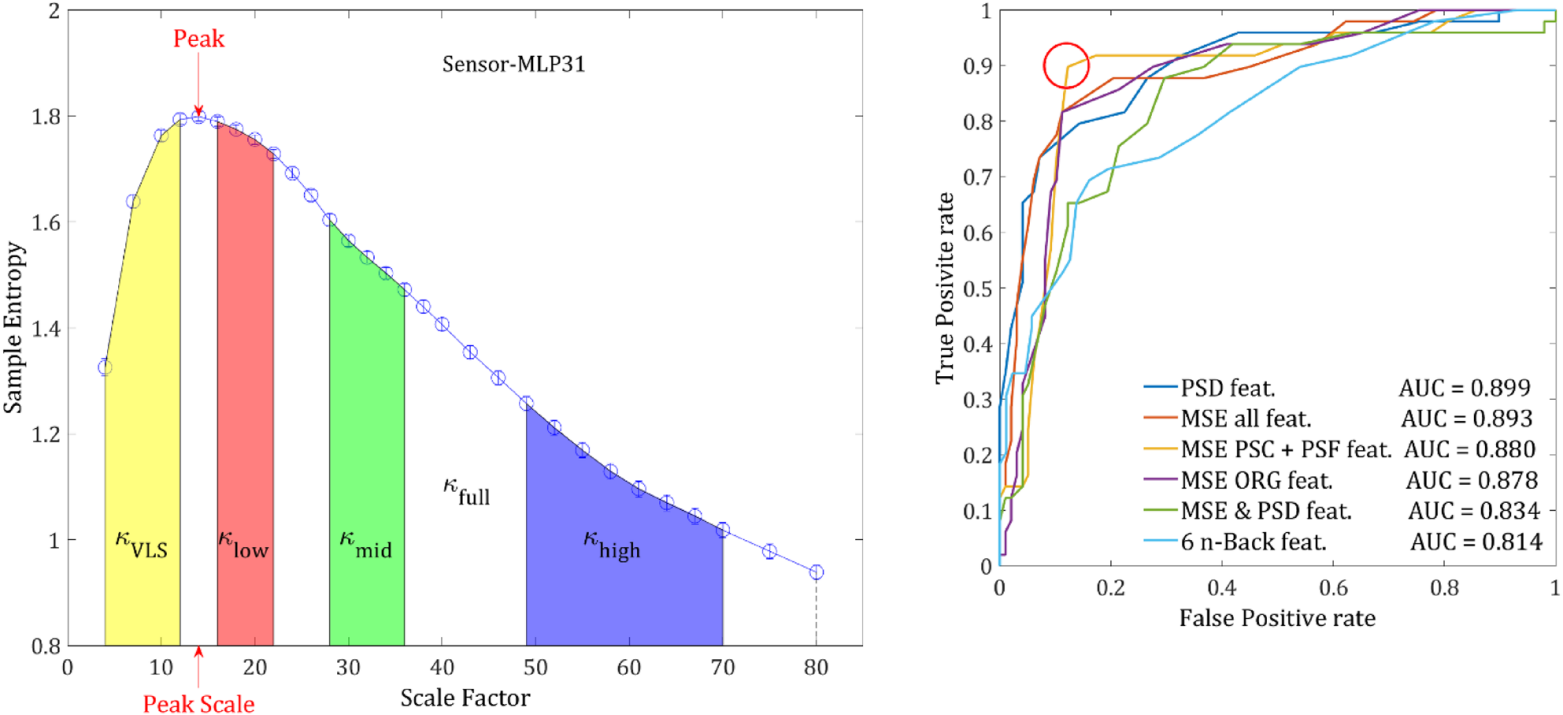
classification. (**a**) features extracted from a single channel. The 7 features that were extracted from each channel: κ values of the VLS range (yellow area), low range (red), mid-range (green), high range (blue) and full range (denoted by the dashed line). The peak is the highest SampEn of each channel and the peak-scale is the scale at which the peak value is found. **(b**) ROC curves ROC yielded by the LDA classification procedure applied on MSE features set (in blue), PSD set (orange), N- back set (purple) and MSE & PSD merged set (yellow). The red circle indicates an optimal threshold of the merged features set allowing a sensitivity of 0.857 with specificity of 0.215.

First, we extracted from the MSE scores of the original time series (ORG) for each channel of each participant the following features (Fig. 7): 5 κ values derived from 5 scale regions full, high, mid, low, and VLS (see Table 1), peak value (the highest SampEn value for a given channel) and peak-scale (which is the scale in which the peak was found). The features were then averaged across channels for each of the 2 ROIs: central and periphery, as was previously suggested according to the anatomical analysis, (Fig. 2b) to produce 14 features from the original time series. For each of these 14 features 4 values were calculated across each sensor ROI: mean, maximum, minimum and the Standard Deviation (SD) for a total set of 56 ($14\times 4$) MSE features for each participant. The same set of 56 features were extracted from the Phase Shuffled Time Series for (PSF) and from the time series of the Non-Linear Component (NLC) for a total of possible 168 features.

To study the classification potential of different feature sets, and to find which features are the most discriminative, we designed a training and validation procedure, based on simple LDA classifiers and utilizing the multi-realization approach (see supplementary Information). To set the chance level of the validation group to 50%, it was truncated to match the number of SCZ patients and controls. For instance, if the validation group had 17 patients and 27 controls then 17 random controls were drawn to match the number of patients.

At first, to get an idea for the minimal number of features needed to reach the best accuracy, we added lasso regularization to the LDA classification procedure and scanned the regularization hyperparameter *λ* between 0.02 and 0.4. The number of significant features dropped rapidly to 8 features at *λ* = 0.12, then to 3 features (*λ* = 0.28) and finally converged to 2 features at *λ* = 0.37. However, the accuracy for 2 or 3 features was considerably lower (73% ± 5%), than for 10 features (78.5% ± 5%). Further increasing the number of features did not have any significant effect on the accuracy.

Next, we applied the classification procedure to each of set of features (ORG, PSF & NLC) separately and then to combination of 2 sets and finally all the 3 sets together. The ORG and PSF features yielded very similar results (~ 77%). However, the PSF set converged faster, perhaps as a result of the slightly larger magnitude of PSF features. Although the NLC set alone yielded the poorest results, when combining it with ORG or PSF sets the accuracy increased by 3–4%. This result strengthens the findings that the NLC component contains additional information. The combination of PSF & NLC and ORG & NLC yielded very close results, which could be expected since the magnitudes of ORG and PSF features are of the same order and about 10 folds larger than the magnitude of NLC. There is practically no increase when combining all 3 sets together. In terms of Receiver Operating Characteristic (ROC), all the feature sets except for the NLC yielded an AUC (Area Under the Curve) between 0.8 and 0.9 (Table 2 & Fig. 7). The joint of all 3 feature sets yielded the slightly highest AUC of 0.899. However, the set of PSF & NLC yielded the most balanced ROC curve (Fig. 7), allowing a considerably better optimal threshold. The sensitivity and specificity were 0.898 and 0.122, respectively. The results are summarized in Table 2.

**Table 2:** Classification results of different subsets.

Set	Num. of features	Max accuracy (SD)	Selected features	AUC
ORG	56	77.1 (5.7)	19	0.878
PSF	56	77 (5.5)	8	0.8433
NLC	56	69.4 (6.5)	9	0.744
PSF + NLC	112	79.8 (4.9)	14	0.888
ORG + NLC	112	80 (5.5)	17	0.880
ORG + PSF	112	81.2 (5.5)	17	0.855
ORG + PSF + NLC	168	81.3 (5.5)	20	0.893
PSD	240	79.2 (5.7)	19	0.834
PSD + PSF + NLC	352	80.3 (6)	18	0.876
n-back	6	77.1 (6)	6	0.814
Minimal subset	28	76.2 (6)	14	0.828

The maximum accuracy is the level of correct classification of the validation group in precents, in parentheses is the standard deviation across the realizations. The selected features are the number of features selected for the maximal accuracy. AUC is the corresponding AUC-ROC value for the selected features.

When trying to select a minimal subset of the most discriminative features, we noticed a considerable multi-collinearity among different features and particularly between the mean and max values of the same features. After resolving most of the collinearity issues (see supplementary Information) we converged to a subset of 14 features, each of them is allowed to interchange between the mean and max values. The accuracy of the minimized subset came close to the original set yielding 76.2 ± 6% and AUC = 0.828 (Table 2).

### Comparison with power spectrum density based classification

To examine whether the MSE analysis has an advantage over conventional power spectrum features (in terms of classification power), we applied a similar LDA classification procedure to compare the discriminative power of MSE derived features to PSD features. From each channel we extracted the conventional power bands δ, θ, α, β, γ and averaged their magnitude across the 6 conventional ROIs in EEG/MEG analysis: Frontal, central, parietal, occipital, left and right temporal (Fig. 2a). We also added the 2 ROIs used in the MSE analysis (Fig. 2b) and the total ROI (all the sensors), for a total of 9 ROIs and accordingly 45 features (5 bands $\times$ 9 ROIs). For each of these 45 features, 4 values were calculated across each sensor ROI: mean, max, min and the standard deviation for a total of 180 values representing the power spectrum of each participant. Next, we performed the same classification procedure as for MSE features. The results were similar to MSE and converged to accuracy of 77.8 ± 5.3% and AUC = 0.834. The most discriminative features were related to δ and α power bands in agreement with the existing literature^3^. Lastly, we tried to merge the features of MSE and PSD to see whether the joint discriminative power would increase. The best 20 MSE features were added to the best 20 PSD features to form a joint one set of 40 features. The same LDA classification procedure was applied to the joint set. Both accuracy (79.7 ± 5.4%) and AUC (0.876) slightly improved converging to almost the same values as MSE features alone.

### Comparison with a cognitive task (n-back)

The results of each n-back block are given by two variables: accuracy and reaction time (RT). Since each participant was asked to perform 3 blocks (*n* = 0, 1 & 2), this results in 6 variables representing each participant’s performance: Score0, Score1, Score2, RT0, RT1 & RT2 for *n* = 0, 1 & 2, respectively. After discarding participants who did not perform or did not complete the task as required, we were left with 129 participants in total (42 SCZ patients and 87 controls). Performing the same classification procedure as for MSE and PSD features yielded very similar results (Table 2). When comparing the discrimination of a single feature, the n-back feature gives a slightly but not significantly better result. When comparing three n-back features, the results were equivalent to that of 10–15 MSE or PSD features. The most discriminative feature was Score2, followed by RT2 or Score1, the remaining 3 did not contribute much to classification.

### XG boost classification

To make sure we thoroughly explored the discriminative power of the MSE method, we applied a different method of classification, the XG boost method that is based on a decision tree. We applied it to the representative subset of the 28 features. The results were quite similar, with accuracy around 76% independent of the initial parameters, and the best AUC was 0.791.

## Discussion and conclusions

The MSE method is an extension of the sample entropy method across different time scales. Our analysis showed that sample entropy across a broad range of time scales of SCZ patients is higher than the sample entropy found in controls. Since higher entropy indicates a less orderly system, it might imply that MEG/EEG time series of SCZ patients are less synchronized than time series acquired from healthy participants (Fig. 1). The difference is significant and exhibited a distinct spatial pattern across the scalp. All the participants had lower SampEn (hence more orderly signal) in posterior frontal, parietal, and anterior occipital sites (Figs. 2 and 3). These areas were also the areas in which the difference in terms of ΔSampEn between SCZ patients and controls was the most significant.

Further analysis suggested that most of the difference originates from the amplitude of the signal and peaks in scales that might be attributed to the *δ, α* and *β* power bands (Fig. 1d). These findings are for the most part, in agreement with the existing literature, according to which different cognitive impairments in schizophrenia are manifested as abnormality in the *α* and *δ* bands^4^. However, a closer look at the phase component of the SampEn reveals that it contains more than just random noise. Even though the difference in the phase component of the SampEn is not significant for most of the channels, it has a clear spatial pattern (Fig. 6) yielding a significant difference when summing across the peripheral ROI (Fig. 2b). Furthermore, when correlating between SampEn (in terms of *κ*) of low and high scales on the phase shuffled time series we attained a low negative correlation in the central ROI and much higher negative correlations in the periphery. This phenomenon implies a complementary relationship between the amplitude and phase components.

Even though the difference of the phase component is significant, it did not contribute to classification due to its small magnitude (in terms of ΔSampEn) in comparison with the linear (amplitude) component. Due to the noisy nature of the MEG and EEG technologies we believe it will require much larger groups of participants to utilize the phase (nonlinear) features for classification.

There are two major issues that should be addressed. First, MSE analyses from studies on other diseases involving cognitive impairment such as Alzheimer and ASD^37–41^, yield results that somewhat resemble each other. The common feature of all these studies was a higher SampEn (hence less orderly signal) in patients with cognitive impairment in comparison to healthy controls. These findings suggest a possibility that the differences in the SampEn are a more general manifestation of the impaired cognitive process, rather than a specific characteristic of SCZ.

The other issue is the robustness of the results. As was already discussed in the introduction, there is high variability in findings among studies based on complexity estimators (sometimes, even within the same study). A great deal of this variability is attributed to factors like medication effects, symptomatology, age, and even social background^61,62^. Due to obvious logistic and regulatory difficulties, most of the studies contain rather a small group of subjects and include no more than one or two medical centers. This makes the studies very susceptible to biases emerging from social background, age, differences in diagnosis and treatment approaches between medical centers.

The number of subjects in our study was substantial (*n* = 49). Yet, it was not fully representative of the SCZ patient’s population. The age distribution was quite narrow (about of 33 ± 2 years for both men and women), and the data were acquired from a single medical center with all patients under antipsychotic medication. While SampEn estimators are generally considered robust^31^, the MSE results of Takahashi et al.^42^ were found to be susceptible to antipsychotic drugs. Our results, though, are in good agreement with Takahashi’s post-treatment reported values.

Nevertheless, our results are promising. The analysis showed that the sample entropy of SCZ patients is significantly higher than the sample entropy found in controls across two orders of magnitude of temporal scales. Specifically, a higher sample entropy was observed across specific time scales related to the δ, α and β power bands in agreement with existing literature. Furthermore, we show that the phase component of the MSE metric contains additional information that is not manifested in the conventional power spectral analysis. However, this component is rather small and does not make a decisive contribution to the efforts of finding a reliable bio-marker for schizophrenia.

Future research should aim to expand the scope and depth of understanding in several key areas. First, utilizing larger datasets would enhance the statistical power and generalizability of the findings, allowing for more nuanced analyses of the complex neural signatures associated with schizophrenia. A longitudinal approach, tracking individuals with schizophrenia over time, would offer invaluable insights into the progression of the disorder and the impact of treatment interventions, including medication effects on neural complexity. Exploring MSE in stimulus-evoked data may provide additional insights which may not be obtained using resting-state data. Another important direction would be reproducing these analyses in EEG data. This would help bridge the gap between research and clinical practice, as EEG is more accessible and feasible in clinical settings. Finally, directly correlating the MSE measures with the spectrum of positive and negative symptoms of schizophrenia could reveal specific neural correlates of symptomatology, paving the way for targeted therapeutic strategies and personalized medicine approaches. These future directions could significantly advance our understanding of schizophrenia and open new avenues for diagnosis, monitoring, and treatment.

### Supplementary Information

Supplementary Information.

## Data Availability

The data analyzed in this study were collected at the MEG facility of the NIH for a previously published study [Shriki et al., 2013]. The data are available from O.S. (shrikio@bgu.ac.il ) on reason.

## Code availability

The code which applies an MSE analysis to resting state MEG/EEG data is available on github: https://github.com/ejwolfson/MSE-cpl-code-public.git
