# Supplementary material for: Multi-scale entropy assessment of magnetoencephalography signals in schizophrenia
Source: Sci Rep. 2024 Jun 25;14:14680. doi: 10.1038/s41598-024-64704-2 (PMC11199523; doi:10.1038/s41598-024-64704-2)
Supplement: Supplementary file 1 — Supplementary Information. [file 41598_2024_64704_MOESM1_ESM.pdf]

Supplementary Information

Phase shuffling analysis

To generate the surrogated data, we employed a method suggested by Prichard & Theiler. This method enables the creation of a distinct time series while preserving the power spectrum and autocorrelation function of the original time series. This preservation is achieved by independently rotating each frequency component of the power spectrum (Fig S1) by a random degree between (0, 2π). Subsequently, an inverse Fourier transform is performed to reconstruct the phase-shuffled time series.

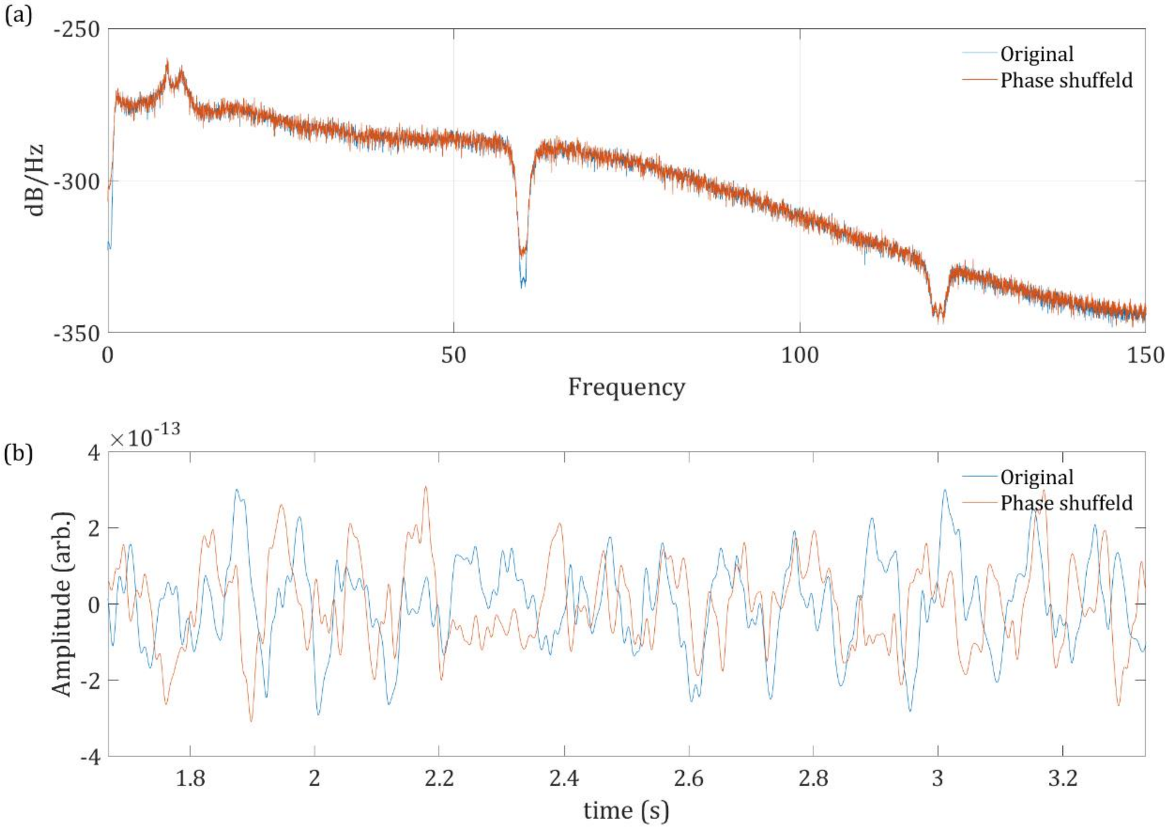

Figure s1: generation of the surrogated data. (a) The power spectrum density PSD of the original (blue) and the surrogated (phase shuffled) signals. (b) A short time window of the original and surrogated time series.

If the original time series was generated by a linear process and can be entirely explained by a linear model, then further nonlinear analysis should not yield any significant differences. However, if the results of the nonlinear analysis for the

original and phase-shuffled time series significantly differ, it indicates the involvement of nonlinear processes in the generation or the original time series.

Robustness check

In order to show robustness of the effect to other possible parameters. And especially since the groups were not balanced sex wise. We tested the same effect on sex and handedness. To do so, we performed a t-test on the same effect size (as defined in equations 6 &7) across the full scales range (4 – 80 SF). First, we preformed the test on each channel separately, then across the averaged anatomical areas (as defined in figure 2) in which the effect size was significant: Total, Central, Parietal and Occipital, to which we added the 2 areas suggested by us according to effect size mapping. Table s1 summarized the results.

Table s1: t-test for sex, handedness. The channels row is the number of channels in which the effect reached the significance level of $p < 0.05$, for each test and group. The values in the κ rows for SCZ, sex and handedness tests, are the p-value for the corresponding test and group.

Test	SCZ	Sex			Handedness		
Group	All subjects	All subjects	Controls	SCZ	All subjects	NC	Controls
Channels	124	46	28	0	0	0	0
κ Total	0.054	0.062	0.141	0.761	0.477	0.679	0.295
κ Central	0.006	0.052	0.298	0.526	0.62	0.818	0.24
κ Parietal	0.004	0.097	0.711	0.433	0.581	0.656	0.31
κ Occipital	0.028	0.424	0.742	0.927	0.952	0.933	0.586
κ Center	0.007	0.072	0.520	0.502	0.704	0.805	0.327
κ Periphery	0.495	0.11	0.7	0.96	0.42	0.647	0.396

Classification procedure

We designed the following multi-realization procedure: for each realization all the participants were divided into a training group and a validation group, with a 70/30 ratio (103 and 44 participants in the training and validation groups correspondingly). To set the chance level of the validation group to 50%, it was truncated to match the number of SCZ patients and controls. For instance, if the validation group had 17 patients and 27, controls then 17 random controls were drawn to match the number of patients. Next, the LDA classifier was trained and validated for each feature separately. The most discriminative feature was pulled out of the features pool and "fixed", moved to a subset of selected features. Next, the classifier was trained for each of the remaining features together with this subset of the features selected up to that point. Again, the feature which yielded the most improvement was "fixed". This procedure continued until 30 features were selected. This provided a ranking between 1-30. After completing one realization, new training and validation sets were drawn from the same data and the procedure repeated to get a new set of significant features. The initial goal was to average the feature rank across many realizations to get an idea of which features are the most discriminative. We applied the following procedure for each set of features, with 300 randomly generated realization.

Correlations among features and minimal subset

When trying to select a subset of the most discriminative features, we noticed that there are few groups of features that tend to interchange similar ranks among themselves. Then once, one of the interchanging features has been selected, the remaining features would drop down in ranking and barely have any effect on the classification. This phenomenon strongly indicates a multi-collinearity among different features. For instance, especially high correlation was found between the mean and max values of the same features (e.g. $\bar{\kappa}_{high}$ & $(\kappa_{high})_{max}$) which tend to interchange between themselves among the selected features, another group of values that tend to interchange are the κ values κ_{full}, κ_{full}^{center}, κ_{high}^{center}, $\kappa_{high}^{periph.}$.

The first step to resolve the multi-collinearity was to assess the correlation between the mean, max, min and SD values of the same features. The correlations between

mean and max were high (R $>$ 0.9) for almost all the features. In contrast, the correlation between mean and min was somewhat lower (R $\sim$ 0.7 on average) and there were practically no correlations between each of the 3 and the SD value. Since in the previous classification we observed that min and SD were barely contributing to the classification if at all, we did not consider them further. For the sake of simplicity and to attain some new insights out of the classification, we decided to focus on the mean values of the features, therefore the max values were also dismissed.

Next, looked at the correlation between the same features from different sets (e.g., the correlation between κ_{high}^{center} in the ORG set and κ_{high}^{center} in the PSF set). As expected, the correlation between the same features in the ORG and in PSF were quite high, with average R $=$ 0.77 $\pm$ .16, with only one feature with correlation below 0.6 (peak$^{peri.}$ with R $=$ 0.4). Also, not surprising was the negative correlation R $=$ -0.22 $\pm$ 0.33 on average between features in PSF and the same features in NLC, while the average correlation between ORF and NLC were somewhere in between and with R $=$ 0.2 $\pm$ 0.25. The most negative correlation was found in κ_{low} and κ_{VLS} in the in the periphery, R $=$ -0.58 and -0.35 respectively, and a somewhat less negative in the center (-0.41 and -0.24 for κ_{low} and κ_{VLS} respectively). This result is in good agreement with the analysis of the Non-Linear Component (Fig. 6, in the main text).

Next, we grouped the features of each set, into 3 natural families and correlated all the features within each of the families in attempt to find highly correlated features. Naively, we grouped the features into the 3 natural families:

κ features – included 10 features (5 scale regions $\times$ 2 ROIs).

Peak features – included 2 features (2 ROIs).

Peak scale features – included 2 features (2 ROIs).

As expected, the correlations among the peak-scale family were high. The average R-scores between features extracted from the original signals was 0.95 and 0.92 from the PSF signal. The correlations between corresponding features extracted from different time series (PSF and ORG) were 0.9 and 0.82 for central and peripheral ROIs correspondingly. Therefore, this family was reduced to one representative feature

that was later shown to be the most discriminative one. The correlations of the peak features were considerably lower. The correlations between the center and periphery were 0.47, 0.44 and 0.83 for the ORG, PSF and NLC sets correspondingly. For the κ features, the picture was somewhat more complex. The values $\kappa_{\text{full}}, \kappa_{\text{high}}, \kappa_{\text{mid}}$ yielded high correlation among themselves ($R > 0.92$) for the same ROI and features set (see table s2). Except for Periphery of the ORG set where the correlation was a bit lower (R=0.83). In the PSF set, there were also high correlation between the 3 values in the center and in the periphery ($R > 0.91$). The κ_{low}, κ_{VLS} features had considerably lower correlations between ROIs ($0.53 < R < 0.77$). The correlation between features sets for κ_{low} ranged from strong positive correlation between ORG and PSF (R = 0.87 & 0.74 for center and periphery correspondingly), to negative correlation between PSF and NLC (R = -0.42 & -0.58), the correlation between ORG and NLC were low (R = 0 & -0.19) as expected. These results are also in good agreement with the previous analysis (Figs. 5 & 6, in the main text).

Following this analysis, features from the same family that were highly correlated ($R > 0.8$) among themselves, were grouped together. For each group, a single representative feature was chosen by performing LDA classification on each individual feature and choosing the most discriminative one. Since the combination of PSF and NLC features yielded the most effective classification (Table 3, in the main text), we focused on feature groups from these two sets.

Eventually the initial set of 168 features was reduced into a subset of 14 representative features. We performed the same classification procedure on the reduced subset of the 14 features. The accuracy was 71 ± 5 % and the AUC was 0.772, which is considerably lower than classification with a full set of 112 features ($80.2 \pm 5.6\%$ and 0.87 respectively). However, once we add back the max value of each of the 14 features for a total of 28 features. The accuracy almost leveled out yielding 76.2 ± 6 % and AUC = 0.828 (Table 3, in the main text). This result strengthens the suggestion that the reduced subset is indeed representative.

Table s2: Features grouping. Each group is presented in a different color. In white are features which has no groping (i.e., don't have R > 0.75 with any other feature). For groups of two features the correlation R is presented, for larger groups the minimal R correlation among all possible correlations is presented.

<table>
<tr><td>Feature</td><td colspan="2">ORG</td><td colspan="2">PSF</td><td colspan="2">NLC</td></tr>
<tr><td>Location</td><td>Center</td><td>Periphery</td><td>Center</td><td>Periphery</td><td>Center</td><td>Periphery</td></tr>
<tr><td>Peak</td><td></td><td></td><td>f_1</td><td>f_2</td><td colspan="2">f_3 R =0.83</td></tr>
<tr><td>Peak-scale</td><td colspan="4">f_4 (R > 0.82)</td><td colspan="2">f_5R = 0.79</td></tr>
<tr><td>κ_{full}</td><td rowspan="3">R > 0.93</td><td rowspan="3">R > 0.83</td><td colspan="2" rowspan="3">f_6 (R > 0.91)</td><td rowspan="3">f_7 (R > 0.97)</td><td rowspan="3">F_8 (R > 0.92)</td></tr>
<tr><td>κ_{high}</td></tr>
<tr><td>κ_{mid}</td></tr>
<tr><td>κ_{low}</td><td></td><td></td><td>f_9</td><td>f_{10}</td><td colspan="2">f_{11} (R = 0.77)</td></tr>
<tr><td>κ_{VLS}</td><td>R=0.95</td><td>R=0.97</td><td>f_{13} (R=0.95)</td><td>f_{14} (R=0.97)</td><td colspan="2">f_{12} (R = 0.75)</td></tr>
</table>
